# Signal Quality of Reflective-Mode Photoplethysmograms Across Anatomical Sites

**DOI:** 10.3390/s26102986

**Published:** 2026-05-09

**Authors:** Federica Ricci, Cecilia Vivarelli, Eugenio Mattei, Giovanni Calcagnini

**Affiliations:** 1 Department of Industrial, Electronic and Mechanical Engineering, Roma Tre University, 00146 Rome, Italy; 2 Department of Cardiovascular, Endocrin-Metabolic Diseases and Ageing, Italian National Institute of Health, 00161 Rome, Italy; eugenio.mattei@iss.it (E.M.); giovanni.calcagnini@iss.it (G.C.); 3 National Center for Artificial Intelligence and Innovative Health Technologies, Italian National Institute of Health, 00161 Rome, Italy; cecilia.vivarelli@iss.it

**Keywords:** reflective-mode photoplethysmography (PPG), signal quality indices (SQIs), optical sensors, anatomical site mapping, heart rate (HR) estimation, pulse transit time (PTT)

## Abstract

**Highlights:**

**What are the main findings?**

- Skewness, Perfusion Index and Kurtosis were the most informative signal quality indices for distinguishing reflective PPG performance across anatomical sites.
- Several non-conventional anatomical sites (lips, nose, and temple) yielded signal quality comparable to the finger, while the wrist consistently showed the poorest performance.

**What are the implications of the main findings?**

- Non-conventional anatomical sites (lips, nose, temple, ear) represent promising alternatives for reflective-mode PPG acquisition in wearable sensing.
- GREEN wavelength reflective-mode PPG, combined with Skewness and Perfusion Index, may provide accurate heart rate estimation from non-conventional sites, supporting improved multisite wearable monitoring strategies.

**Abstract:**

Reflective-mode photoplethysmography (PPG) potentially enables non-invasive physiological monitoring of heart rate and Peripheral Oxygen Saturation (SpO_2_) from virtually any anatomical body site, but its performances are strongly affected by several parameters such as local perfusion, skin temperature, and microvascular bed and tissue optical properties. This study systematically evaluates the quality of reflective-mode PPG signals acquired at the finger, wrist, ear, nose, temple, upper lip, and lower lip, using two commercial PPG sensors. PPG signal quality was quantified via Skewness, Kurtosis, Perfusion Index, and Shannon entropy. Heart rate (HR) and pulse transit time (PTT) were also computed. Skewness and Perfusion Index were the most informative quality indices, revealing the finger as the site with the best signal quality and the wrist as the most challenging location. Several facial regions—including the lips, nose, and temple—showed signal quality comparable to the finger. HR estimation was most accurate using the GREEN wavelength, with the lower lip achieving the lowest error, followed by the upper lip and finger. PTT values reflected physiological differences in pulse propagation, being longest at the finger and wrist and shortest at the lips. These findings highlight the potential of non-conventional anatomical sites as alternatives to the finger and wrist for reflective-mode PPG acquisition.

## 1. Introduction

Photoplethysmography is a simple, low-cost, and non-invasive optical technique used to detect the pulsatile changes in blood volume within the microvascular bed of tissue [1,2,3]. Since its early development, PPG has become a core technology in modern wearable devices, enabling the estimation of peripheral oxygen saturation (SpO_2_), heart rate (HR), pulse wave morphology, and vascular parameters.

PPG works by emitting light (green, red, or infrared) into the skin using LEDs and measuring the amount of light reflected or transmitted using a photodetector. Transmitting mode, where light passes through the tissue, requires source and emitter on opposite sides, and it is mainly obtained from the extremities of the human body, such as fingers, toes or earlobes; reflective mode, where the photodetector captures backscattered light from the same side as the source [1,3], may be virtually used in any anatomical site, potentially enabling wearable monitoring solutions in extreme environments (e.g., diving, space, low temperature).

While transmission-mode PPG typically offers higher signal quality due to clearer optical paths, reflective-mode PPG is far more versatile for wearables and body mapping. However, reflective-mode PPG is also more sensitive to confounders such as skin curvature, optical coupling, and local perfusion [4]. Comparative analysis has shown that the two modalities differ in penetration depth, susceptibility to motion, and robustness under vasoconstriction [5], with early studies noting the higher vulnerability of peripheral reflective sites during low-perfusion states [2].

Despite the technological improvements in modern integrated PPG modules—offering multi-wavelength illumination, compact form factors, and low power consumption—signal reliability remains highly dependent on anatomical site and physiological conditions [6]. Motion artifact, vasoconstriction, variable sensor-skin contact, and wavelength-dependent penetration depth can significantly deteriorate signal quality [7]. Several studies have demonstrated that peripheral vasoconstriction reduces PPG amplitude and affects pulse transit time (PTT) dynamics, underscoring the limitations of peripheral optical sensing [8]. Similarly, during hypothermic conditions, finger PPG signals show a higher reduction compared to only minimal reductions in ear-canal PPG, emphasizing how anatomical site and perfusion status critically impact signal reliability [8].

Multiple comparative studies have evaluated the performance of PPG signals across various body sites, wavelengths, and sensor configurations. Findings consistently show that measurement accuracy varies considerably with anatomical site: when using reflective sensors, the fingertip and forehead often yield the most reliable HR and SpO_2_ estimates, though they exhibit different susceptibilities to motion and respiratory modulation [9,10]. These observations motivate the need for systematic body site mapping using reflective PPG, especially with the new generation of wearable-grade optical sensors.

Reflective-mode PPG has been investigated at well-established sites, such as the finger and wrist [5], particularly driven by the use of smartwatches. Alternative anatomical sites such as the ear, temple, and nose [6,9] have started to receive attention as suitable monitoring sites for the red and infrared signals, and preliminary evidence has been collected on signal quality and feasibility. Few data comparing red, infrared, and green signal quality from these alternative sites have been published [8,9,10].

Numerous signal quality indices (SQIs) have been proposed to discriminate cleanliness from noisy PPG segments. A set of SQIs has been introduced and evaluated in the literature—including skewness, kurtosis, entropy, perfusion index (PI), and others—showing that statistical shape-based metrics such as skewness and kurtosis can effectively distinguish reliable PPG segments from corrupted ones [11]. These findings align with broader reviews indicating that motion artifacts substantially distort PPG morphology and features, which has motivated the development of feature-based or machine-learning-based quality classification approaches [12].

In recent years, new integrated reflective-mode PPG sensors have gained prominence due to their compactness, low power consumption, multi-wavelength capabilities, and suitability for wearable devices [13].

Motivated by prior evidence demonstrating the diagnostic value of PPG morphology analysis [14] and the promising potential of SQIs for robust signal assessment, this study aims to provide a systematic and quantitative evaluation of reflective-mode PPG across multiple anatomical sites.

The main objective of this work is to assess and compare the signal quality of reflective red, infrared, and green PPG sensors positioned across the following anatomical sites: finger, dorsal wrist, ear (specifically the inner part of the tragus region), temple, nose, inner upper lip, and inner lower lip. In addition, for each anatomical site, we evaluated the performance to detect HR and the impact on the PTT values.

## 2. Materials and Methods

Two reflective-mode PPG sensors were used: MAX30101 (Analog Devices, Wilmington, MA, USA—RED, IR, and GREEN LEDs) and ADPD144RI (Analog Devices, USA—RED and IR LEDs). A single-lead ECG (Cardionica, Cardionical srl, Roma, Italy) and a SpO_2_ monitor (MIROxy, Medical International Research, Roma, Italy) were used as reference. A temperature sensor was also used (MAX30205, Analog Devices, USA) to measure both ambient and skin temperature.

### 2.1. Experimental Setup

An acquisition system was developed to synchronously record ECG, the multi-wavelength PPG, and the skin and ambient temperature. Data were collected from healthy volunteers at multiple body locations following a randomized protocol. Subsequently, PPG SQIs, HR, and PTT were computed and compared to the finger.

**Figure 1:**
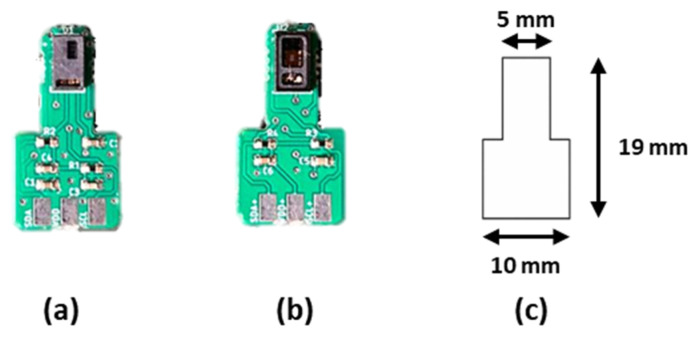
(**a**) ADPD144RI, with red and infrared LEDs, (**b**) MAX30101, with red, infrared, and green LEDs, (**c**) the dimensions of the PCB.

The two PPG sensors, shown in Figure 1, include the optical elements, internal LEDS, photodetectors, and low-noise electronics; the sensors were mounted on a printed circuit board (PCB), designed to be compact and easy to handle during placement at different anatomical sites. Although both sensors integrate the main optical components, they differ in design approach, with the MAX30101 being more compact and optimized for wearable applications, and the ADPD144RI offering greater flexibility in configuration and signal management.

**Figure 2:**
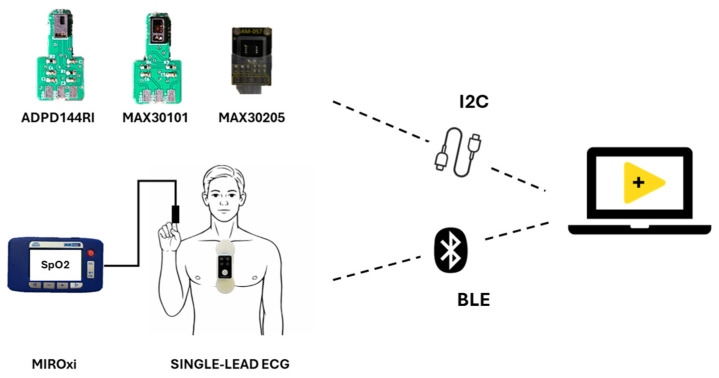
Experimental setup.

Figure 2 shows the setup that is composed of

- A single-lead ECG device, positioned on the subject’s chest.
- Two reflective-mode PPG sensors.
- A digital temperature sensor for ambient and skin temperature monitoring.
- A commercial transmission pulse oximeter, used to monitor SpO_2_.

All optical and temperature sensors were interfaced via a NI USB-8451 I2C data acquisition module (National Instruments, Austin, TX, USA), which handled communication. A custom-designed circuit enabled simultaneous operation of the ADPD144RI, MAX30101, and MAX30205 sensors, providing appropriate supply voltages and LED driving conditions according to each device’s electrical specifications. ECG data were transmitted to a laptop via Bluetooth Low Energy (BLE) using an nRF52840-Dongle (Nordic Semiconductor, Trondheim, Norway). The single-lead ECG device was used as a ground truth for HR estimation (RR-peak) and for PTT estimation (i.e., the time delay between RR-peak on ECG and systolic peak on PPG). Attention was paid to guarantee the synchronization of all signals.

All acquired signals were visualized in real time through a custom LabVIEW-based graphical interface (National Instruments, USA), allowing continuous monitoring of signal quality and sensor status during the acquisition session. In addition, the interface enabled full configuration of the sensors by programming internal registers, including LED driving current, sampling frequency, and sample averaging settings, as well as the synchronization between the ECG and PPG signals.

### 2.2. Data Collection Protocol

Data were collected from 30 healthy volunteers (15 males and 15 females), between 24 and 57 years old, with resting SpO_2_ between 96% and 99% at the time of measurement. Informed consent was obtained from all subjects involved in the study.

All recordings were carried out in an indoor environment with an ambient temperature between 22 and 29 °C. Participants remained seated throughout the experiment and were instructed to maintain relaxed, regular breathing while minimizing movement to reduce motion artifacts.

The transmission pulse oximeter sensor was placed on the second finger of the right hand. The single-lead ECG device was placed on the chest using its adhesive electrodes. Ambient and skin temperatures were measured at the beginning and at the end of each session.

**Figure 3:**
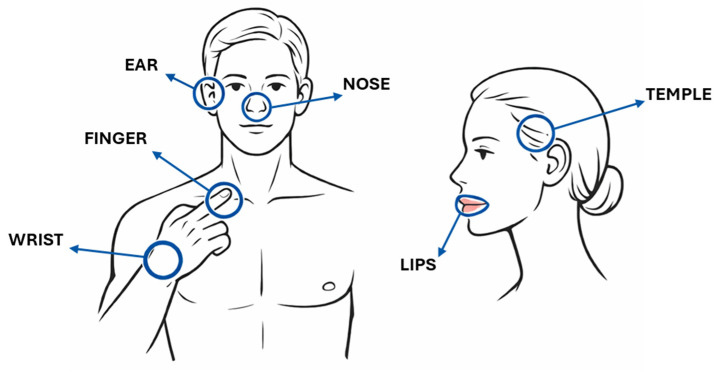
Anatomical locations used for reflective-mode PPG measurements.

Each participant was measured through reflective-mode PPG sensors in seven anatomical sites shown in Figure 3: second fingertip, dorsal wrist, tragus region of the ear, nose, temple, inner lower lip, and inner upper lip.

All participants were asked to keep the PPG sensor in place and avoid excessive force, and, if necessary, slight repositioning was performed to optimize waveform quality before starting the session. The PPG sensor gain was optimized to maintain a consistent dynamic range across subjects and anatomical sites. For each site, 60 s of each PPG sensor was recorded in a random order. Similarly, the seven anatomical sites were tested in a randomized sequence, ensuring that no systematic ordering may have influenced the results. Randomization was performed individually for each subject, and the full randomized sequence was completed for the first sensor before repeating the procedure with the second sensor.

### 2.3. Signal Processing and Statistical Analysis

Computation of PPG SQIs and estimation of HR and PTT were performed for each sensor and anatomical site. A 20s PPG, artifact-free, segment was analyzed together with the corresponding synchronized ECG trace. All signal processing and analysis procedures were performed using LabVIEW 2019.

#### 2.3.1. Computation of PPG SQIs

The raw PPG signals were first band-pass filtered using a 4th-order Inverse Chebyshev digital filter [15] with a low cut-off frequency of 0.5 Hz, a high cut-off frequency of 8 Hz, and a minimum stopband attenuation of 20 dB. This filtering step removed baseline wander and high-frequency noise while preserving the cardiac component of the waveform. Since the PPG signals were acquired in reflective mode, the filtered signals were inverted to obtain the conventional PPG morphology with positive-going systolic peaks.

The filtered PPG signals were then segmented into non-overlapping 2 s windows (window length of 2 s with a step of 2 s). The 2 s window was chosen according to Elgendi [11]. Within each window, the SQIs were computed, and, for each sensor and anatomical site, the final SQI value was obtained by averaging the values across all windows of the 20 s segment.

The four PPG SQIs considered in this study—Skewness, Kurtosis, Perfusion Index, and Shannon entropy—were selected based on the established literature, as they have been shown to effectively capture different aspects of PPG signals’ morphology, pulsatility, and complexity [11].

The Skewness of the signal quantifies the asymmetry of the amplitude distribution around its mean:

(1) $$skewness=\;\frac{1}{N}\sum_{i=1}^{N}{\left[x_{i}-\;\frac{\hat{\mu_{x}}}{\sigma }\right]}^{3},$$

The Kurtosis of the signal measured the sharpness of the amplitude distribution:

(2) $$kurtosis=\;\frac{1}{N}\sum_{i=1}^{N}{\left[x_{i}-\;\frac{\hat{\mu_{x}}}{\sigma }\right]}^{4},$$

The Perfusion Index (PI) was computed as the ratio between the pulsatile (AC) and non-pulsatile (DC) components of the PPG signal. The DC component was defined as the mean value of the raw signals (x_raw_) within the window, while the AC component was defined as the peak-to-peak amplitude of the band-pass filtered PPG signal over the same interval:

(3) $$PI=\;\frac{AC}{DC}\;\times 100=\left[\frac{\left(x_{max}-\;x_{min}\right)}{\left|\bar{x_{raw}}\right|}\right]\times 100,$$

Shannon entropy was calculated using the 20 s filtered PPG segment. The signal was quantized to 16 levels. The normalized histogram of the quantized signal provided empirical probabilities. The Shannon entropy was computed as

(4) $$H\left(x\right)=-\sum p\left(x_{i}\right)log_{2}\left(p\left(x_{i}\right)\right),$$

where p(x) is the estimated distribution of probability of the normalized PPG signal.

The selected SQIs capture complementary aspects of PPG signal morphology and variability. Skewness and Kurtosis describe the statistical distribution of the waveform amplitude and are therefore sensitive to changes in pulse range and peak sharpness. Higher kurtosis values have been associated with well-defined systolic peaks, which are desirable for reliable beat detection and time-domain feature extraction [11]. Shannon entropy reflects the degree of signal complexity and irregularity: lower entropy values are generally associated with more regular and rhythmic waveforms, whereas higher entropy may indicate increased noise or motion-related disturbances [11,12]. Together, these SQIs provide a complementary interpretation of waveform morphology, pulsatility, and irregularity.

Since no reliable estimates of the expected effect sizes for the considered SQIs and anatomical sites were available at the study design stage, the choice of the sample size using an a priori power analysis was not feasible. The sample size was chosen according to previous PPG studies, such as Hartmann et al. [6] (36 subjects) and Elgendi [11] (approximately 40 subjects). An a posteriori power analysis based on the observed effect sizes and variability in the data was then conducted.

For each SQI, the hypothesis that the data is normally distributed was assessed using the Shapiro–Wilk test. Since, for all signals and anatomical sites, the assumption of normal distribution of SQI was not confirmed, all statistical analyses were conducted using non-parametric methods.

Repeated-measured comparisons across anatomical sites were performed using the Friedman test, followed by paired Wilcoxon signed-rank tests for post hoc analysis when a significant difference was observed. Statistical significance was set at *p* < 0.05.

#### 2.3.2. Estimation of HR and PTT

The estimation of HR was performed for all PPG signals (RED and IR for ADPD144RI plus GREEN for MAX30101); however, PTT was computed only for the GREEN signal of the MAX30101, since this wavelength was expected to provide the most reliable signal for PTT estimation.

Since PPG signals were sampled at 50 S/s, whereas the ECG was sampled at 500 S/s, all PPG signals were resampled to 500 S/s using cubic spline interpolation.

For the ECG signals, R-peak identification was performed using a QRS detection based on Pan-Tompkins’s approach [16]. PPG pulse peaks were identified using a threshold-based peak detection algorithm combined with local maximum search within a predefined analysis window.

For both ECG and PPG signals, HR was calculated from the mean value of the beat-to-beat intervals according to the following equation:

(5) $$HR=\;aver\left(\frac{60}{T_{IBI}}\right),$$

T_IBI_ is the inter-beat-interval calculated as t_ECG_(n + 1) − t_ECG_(n) for the ECG signal, and t_PPG_peak_(n + 1) − t_PPG_peak_(n) for the PPG signal, where n denotes the beat index, t_ECG_ = R-wave occurrence time, and t_PPG_peak_ is the occurrence time of the systolic peak of the PPG waveform.

**Figure 4:**
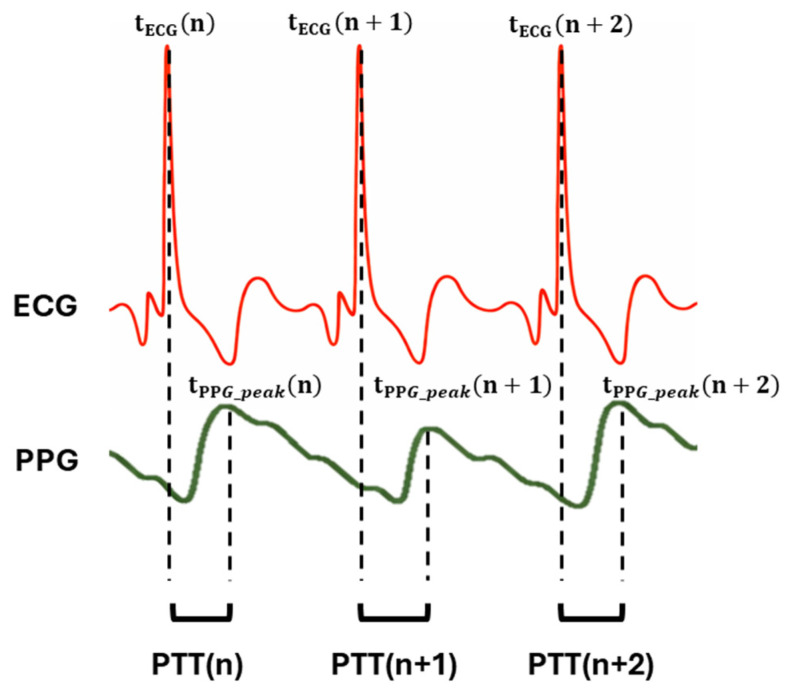
Detection of t_ECG_ and t_PPG_peak_ on ECG and filtered green PPG waveform for PTT estimation.

PTT is defined as the time interval between cardiac electrical activation and the arrival of the pulse wave at a peripheral site. In this study, PTT was calculated using the filtered GREEN PPG signal, as shown in Figure 4, acquired by the MAX30101 sensor as follows:

## 3. Results

In total, 420 recordings were obtained (from seven measurement sites, two sensors, and 30 subjects). None of the subjects reported discomfort or adverse effects during the signal acquisition.

### 3.1. Overall Signal Quality Across Anatomical Sites

In all subjects, PPG signals were collected from all the anatomical sites. Local skin temperature ranged from 24 to 31 °C.

Table 1 and Table 2 summarize the SQIs across the anatomical sites, for both sensors, respectively.

Table 1 and Table 2 report SQI values across anatomical sites for the ADPD144RI and MAX30101 sensors, respectively, enabling a direct quantitative comparison of signal quality trends across both sensors and wavelengths. Statistical significance is reported with respect to the finger and wrist, used as reference sites to highlight relative differences in signal quality across anatomical locations. For both sensors and all wavelengths, the highest values of skewness were obtained at the finger, whereas the lowest ones were detected at the wrist. The differences between finger and wrist were highly significant (*p* < 0.001, Wilcoxon test, for all wavelengths and sensors), confirming the finger as the most reliable site and the wrist as the most challenging location for reflective-mode PPG acquisition. The other sites yielded skewness values lower than finger, higher than wrist, but no significant differences were observed among these sites.

**Figure 5:**
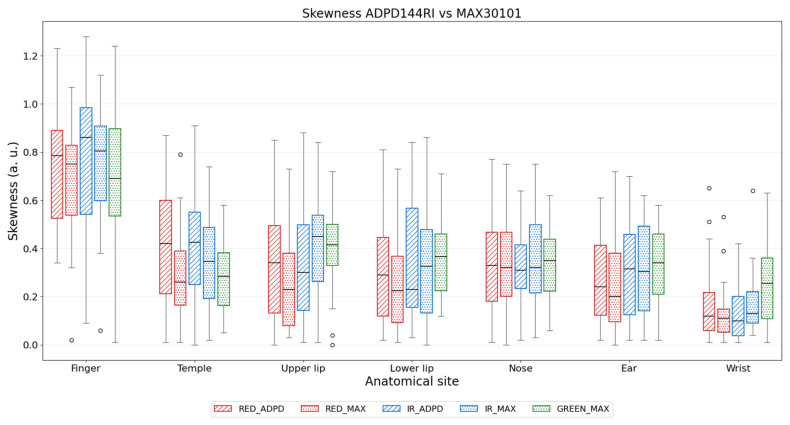
Distribution of Skewness values for RED, IR, and GREEN signals of the ADPD144RI and MAX30101 sensors.

Skewness obtained for the GREEN was not always higher than RED and IR, from the same sensor. Across the different anatomical sites, the skewness of the GREEN signal remained comparable to that of the other wavelengths. When the skewness of the RED and IR signals from the two sensors was compared, no differences were observed: for each site, the two sensors resulted in skewness values with similar ranges (Figure 5).

**Figure 6:**
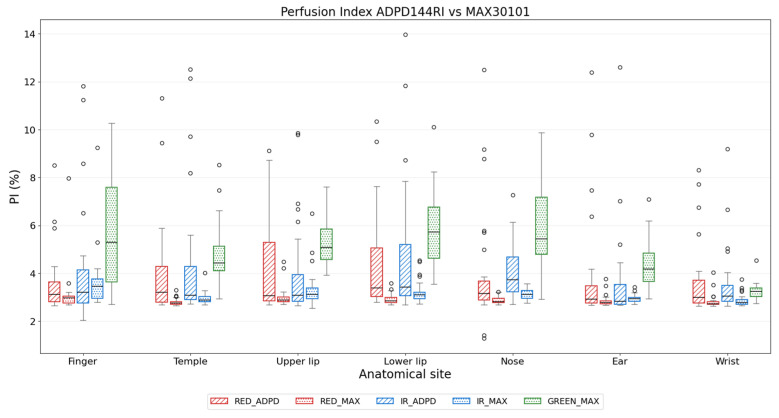
Distribution of PI values for RED, IR, and GREEN of the ADPD144RI and MAX30101 sensors.

Figure 6 shows the PI of PPG signals from the investigated anatomical sites, for both sensors. The PI was lowest at the wrist for all wavelengths and sensors (only RED from MAX30101 at the temple had a slightly lower value). Overall, PI showed weaker statistical differences among sites. Notably, the GREEN signal always yielded the highest population-average PI, regardless of the anatomical site. The PIs of the RED signal from the ADPD144RI sensor were always higher than the MAX30101 ones. This pattern was present at all anatomical sites. The PI values obtained for the RED and IR signals from the MAX30101 showed a lower dispersion compared to the ADPD144RI counterparts, which resulted in a higher statistical difference between sites.

Kurtosis showed significant differences between sites: the highest values were always observed at the finger, followed by the wrist, while the other sites had lower values.

Shannon entropy showed only a few significant differences between sites, regardless of wavelengths and sensors.

Post hoc power analysis showed that the study was sufficiently powered to detect effects of moderate-to-large magnitude, as those observed in skewness, perfusion index and kurtosis (effect size > 0.6, 1 − β values > 0.80) but had limited sensitivity to smaller effects (Shannon entropy). Thus, Shannon entropy was not further used.

A Friedman test was applied on skewness, PI and kurtosis to assess whether at least one anatomical site differed from the others. When this test yielded statistical significance (*p* < 0.05), pairwise post hoc comparisons between sites were conducted using the Wilcoxon test. A “win” is assigned when an anatomical site shows a higher signal quality with a statistical significance in the post hoc comparison (*p* < 0.05).

Table 3 and Table 4 show the result of this analysis for the ADPD144RI and MAX30101, respectively, considering that the “wins” represent the total number of significant wins accumulated by each anatomical site across all pairwise comparisons. For RED and IR signals, the “wins” were added, since, in practice, these signals are collected together to be combined to obtain SpO_2_ values.

### 3.2. Heart Rate Estimation Accuracy

HR estimation accuracy was evaluated by comparing PPG-derived HR values with ECG reference measurements using the percentage error as a metric. This analysis aims to assess whether the selected anatomical sites may represent an alternative for reliable HR estimation using reflective-mode PPG signals.

**Figure 7:**
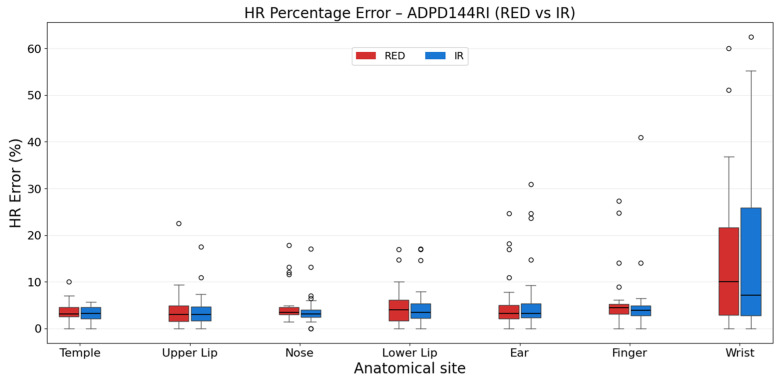
Percentage error distribution between anatomical sites for RED and IR signals of the ADPD144RI.

In Table 5 and Figure 7, the percentage errors for the ADPD144RI are shown. The HR estimation was most accurate at the temple, for both RED and IR signals. In contrast, measurements acquired at the wrist showed significantly larger HR estimation errors due to a balanced presence of missed detected pulse peaks. Upper lip showed a performance only slightly worse than the temple, but with a higher variability.

**Figure 8:**
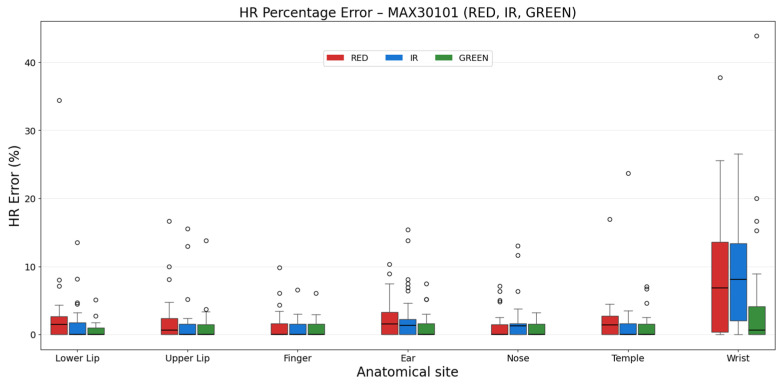
Percentage error distribution between anatomical sites for RED, IR, and GREEN signals of the MAX30101.

In Table 6 and Figure 8, the percentage error values of the MAX30101 are shown. Given that the GREEN signal is widely employed in wearable devices, such as smartwatches, for HR estimation, the anatomical sites in Table 6 were ranked based on the percentage error of the GREEN one.

Overall, the GREEN signal provides the lowest error values at most sites, with the lower lip exhibiting the best performance, followed by the upper lip and finger. The RED and IR signals show comparable performance across sites, generally with higher variability than the GREEN signal. The nose, finger, and temple show the lowest errors for both RED and IR signals. HR estimation from the wrist showed the highest errors for all wavelengths.

The RED and IR signals from the MAX30101 sensors showed a lower error than the ADPD144RI counterparts.

Table 7 shows the mean percentage HR error for different skewness ranges, computed for the GREEN signal. Higher skewness values correspond to lower errors in HR estimation. Skewness values greater than 0.2 resulted in acceptable HR estimation errors.

Table 8 summarizes the mean percentage HR error across different PI ranges, computed from the GREEN signal. In this case, as well, a higher PI is associated with lower HR estimation errors. PI values above 4% yielded acceptable estimation accuracy.

Table 9 summarizes the mean percentage HR error across different Kurtosis ranges, computed from the GREEN signal. Unlike Skewness and perfusion, the highest kurtosis is associated with the highest HR estimation error.

### 3.3. Pulse Transit Time Analysis

**Figure 9:**
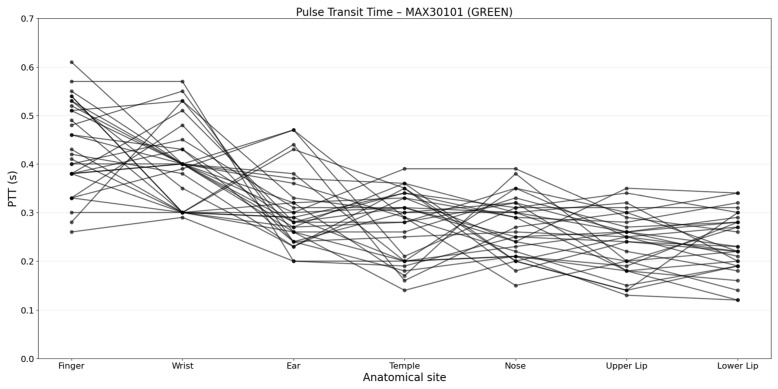
PTT estimation across seven selected anatomical sites; each line represents one subject, illustrating individual PTT variations across locations.

Finally, PTT was investigated across anatomical sites to assess the influence of the anatomical site on the PTT values. PTT analysis was performed using the MAX30101 GREEN signal and the results are shown in Figure 9. The finger and wrist show the highest PTT values, reflecting longer pulse propagation. In contrast, facial sites—especially the upper and lower lip—exhibit the lowest PTT, reflecting shorter propagation times.

Figure 9 shows the PPT distributions across anatomical sites, at the individual subject level. Although absolute PTT values varied substantially among subjects, a consistent pattern was observed across the population, with longer PTT values at peripheral sites such as the finger and wrist and shorter PTT values at facial sites, with a reduction to 50% of the value at the finger. This consistency across subjects supports the physiological robustness of the observed site-dependent trends while highlighting the expected inter-subject variability in absolute PTT values.

### 3.4. Power Budget and Computational Complexity

Power budget was estimated from the manufacturer’s datasheet, assuming typical conditions of use (LED currents, pulse duration, and sampling rate). Under these assumptions, the LEDs average current can be calculated as

(6) $$I_{aver}=I_{VDD}+\left(I_{LED}\times N_{LED}\times PW\times T\right),$$

where I_VDD_ is the internal chip current, I_LED_ is the driving current of each LED, N_LED_ is the number of active LEDs, PW is the LED pulse width, and T is the sampling period.

For MAX30101, given a LED pulse of 411 µs, a pulse number for each sample of 1, a peak LED current of 51 mA for each of the three LEDs, and an internal sampling rate of 400 S/s, the resulting average current of the three LEDs is 25.2 mA, which summed to the internal chip consumption (1.1 mA), yields a total average current of 26.3 mA.

For ADPD144RI, given a LED pulse width of 24 µs, (8 × 3 µs pulses for each sample), a peak LED current of 250 mA for each of the two LEDs, and an internal sampling frequency of 400 S/s, the resulting average current of the LEDs is 4.5 mA, which summed to the internal chip consumption (1.5 mA), yields a total average current of 6.3 mA.

The computational of the proposed SQIs requires the storage of a 20 s data segment for each wavelength. Given a representation of 4 bytes/sample and a sampling rate of 50 S/s, the amount of RAM required is about 12 KB. Skewness, kurtosis and Shannon entropy require the estimation of the distribution of the signal, while PI requires peak detection. In both cases, the computational effort required is well below the resource available in most of the microcontroller available in the market and can be achieved within few hundred ms of computation time.

## 4. Discussion

Reflective-mode PPG may potentially be collected from any part of the body surface. Previous studies have investigated reflective-mode PPG performance across selected anatomical sites, often focusing on traditional locations such as the finger, forehead, or ear and primarily targeting HR and SpO_2_ estimation. In contrast, the present study offers a systematic multisite evaluation that extends the analysis to non-conventional facial regions, including the upper and lower lips, which have received limited attention in prior studies. Moreover, unlike studies relying on qualitative signal inspection or single performance metrics, this work integrates multiple SQIs with HR estimation and PTT values, offering a comprehensive and quantitative framework for anatomical site comparison. The use of two different commercial sensors further strengthens the generalizability of the findings. In this context, non-conventional anatomical sites such as the nose, temple and lips may be potential candidates for monitoring cardiovascular parameters in extreme environments/conditions such as scuba diving, space missions, and sports.

Signal quality was assessed using metrics previously adopted in transmission-mode PPG, namely skewness, kurtosis, perfusion index, and Shannon entropy. For the two PPG sensors used, and for all wavelengths (RED, IR, and GREEN), skewness is the SQI that best differentiated anatomical sites, outperforming the gold standard SQI (i.e., perfusion index). These findings are consistent with the results of Elgendi [11] on transmission PPG, and likely expression of the capability of skewness to capture morphology of the pulse waveform.

Skewness was highest at the finger, while the worst signals were collected at the wrist, confirming that this anatomical site is intrinsically challenging for reflective-mode PPG acquisition, despite being the most widely used site in commercial wearables, such as smartwatches. The upper and lower lips, temple, nose, and ear provided signal quality comparable to the finger. The ranking analysis based on statistically pairwise comparisons confirmed this result: finger ranked first for both sensors, although other sites such as the upper and lower lip, nose, and temple also showed competitive performance. Wrist obtained zero “wins”.

Perfusion index showed a weaker statistical difference across anatomical sites compared to skewness. The lowest perfusion index values were generally observed at the wrist, and higher values were obtained at the finger and at facial sites. A noteworthy finding is that, for the MAX30101 sensor, the GREEN signal systematically exhibited the highest perfusion index values across all investigated anatomical sites. Unlike the skewness, the ranking analysis based on static pairwise comparisons showed a more dispersed pattern, with fewer total wins.

Kurtosis exhibited statistically significant differences among anatomical sites, with the highest values observed at the finger and at the wrist, following the facial sites. This trend suggests that peripheral sites exhibit sharper and more peaked PPG amplitude distributions compared to sites closer to the heart. The ranking analysis showed that kurtosis wins were distributed across multiple sites, which is different from what has been obtained for skewness and perfusion index. This finding suggests that kurtosis mainly captures differences in waveform distribution characteristics.

The comparison between the two sensors for RED and IR signals showed that the perfusion index was higher for signals collected by ADPD144RI, while skewness did not differ between the two sensors. It may be speculated that the ADPD144RI, which does not include a GREEN LED, is optimized for RED and IR signal acquisition.

In all the anatomical sites investigated, the most accurate HR estimation was obtained using the GREEN signals. Finger proved to be the best-performing site for GREEN-based HR estimation, followed by the lower and upper lip. The HR estimation error was correlated with skewness and perfusion index values, which can thus be used as thresholds for HR estimation. Longmore et al. [10] showed that, for RED and IR signals from a MAX30102, the anatomical site with the lowest HR error was the finger, followed by the forehead and the temple, while the wrist displayed the highest error. Our findings confirmed that, for RED and IR signals from the MAX30101, the lowest error was obtained using signals from the finger, and the wrist turned out to be the most challenging site for PPG HR estimation. HR estimation across different kurtosis ranges did not show a clear trend, indicating that this SQI does not provide a direct criterion for identifying PPG signal conditions associated with HR estimation, unlike skewness and perfusion index.

The lips and temple showed similar SQIs and HR accuracy, thus being good candidates for collecting PPG and HR in particular environments: the lips are not significantly affected by external temperatures and may be a good candidate for integration into scuba diving equipment. The temple may be a candidate location for instrumented glasses and masks. Interestingly, Bebout et al. showed that face microcirculation is less affected by peripheral vasoconstriction, and it maintains a more stable microcirculation and more reliable PPG signal [17]. The finger and wrist exhibited the highest PTT values, reflecting longer propagation distances, while the lips showed the shortest PTTs. This behavior is physiologically consistent: sites closer to the heart experience shorter pulse arrival times. In particular, peripheral anatomical sites such as the finger and wrist are supplied by distal arterial branches and involve longer and more compliant propagation pathways, whereas facial sites such as the lips are closer to central circulation and are supplied by branches of the external carotid artery. As discussed in multisite PPG studies, differences in arterial pathways and vascular characteristics across sites are associated with variations in pulse wave arrival time [6]. In terms of absolute values, a large inter-subject variability was observed.

The power consumption analysis highlights a substantial difference between the two sensors. The ADPDP144RI achieves lower average current consumption by employing short, high-current LED pulses, whereas the MAX30101 operates with longer pulse widths, resulting in higher average current. These results confirm that the ADPD144RI configuration is more energy-efficient under the tested conditions, supporting its suitability for low-power wearable and embedded applications, when the GREEN wavelength is not required. The higher average current consumption observed for the MAX30101 is mainly due to its optical configuration, including the additional GREEN LED channel, which turned out to provide the best HR estimation, in all the investigated anatomical sites. Consequently, the higher power budget of the MAX30101 reflects increased sensing functionality rather than reduced energy efficiency, highlighting a design trade-off between power consumption and available physiological information. For both sensors, the power budget is compatible with batteries used in wearable systems. The memory and the computational effort required to compute the proposed SQIs is well below the typical resources available in most microcontrollers in the market.

Other factors beyond signal quality and power budget must be taken into account in order to assess the feasibility of using the investigated anatomical sites for wearable systems. Such factors include susceptibility to movement artifacts, temperature changes and poor perfusion due to clinical conditions, comfort, stability and long-term usability, to name a few.

Even though the study aimed to investigate signal quality as a prerequisite for developing a wearable system, we asked the participant to rate the use of the sensors. No complaints or discomfort was reported by the participants, even during the use on the inner lips. The very small size of the sensors and of the custom-designed PCB allows the sensors to be integrated into clothing, hats, and other accessories.

Interestingly, the use of inner lips to obtain physiological signal was proposed in the Patent Applications [18] for a sensorized scuba mouthpiece, and in [19] for endotracheal tube and cannulas, but no scientific data or ergonomic information is available at the moment. Our data show that the quality of PPG signals obtained from the lip is comparable to the finger.

To our knowledge, the inner lips were not previously investigated as a potential site for reflective PPG.

## 5. Study Limitations

Preprocessing of the PPG signals relied on filter settings and quality indices previously reported in the literature as suitable for transmitting-mode PPG. Whether these choices also represent optimal solutions for reflective-mode PPG was not investigated.

The study was designed as an exploratory, methodological investigation aimed at characterizing reflective-mode PPG signal quality across multiple anatomical sites, rather than testing a predefined clinical effect size. Consequently, non-significant results (e.g., Shannon entropy) should be interpreted with caution, as they may reflect limited statistical power rather than the true absence of an effect.

The present study was conducted under stationary conditions on healthy subjects, and thus it may not provide evidence on the impact of motion, temperature changes, and poor perfusion on the investigated anatomical sites. Signal quality at facial sites such as the lips and temple may be less affected by the body movement than the wrist, but facial movements (e.g., speaking, chewing) are expected to have an impact. A site-specific modeling of the motion artifacts and a dedicated protocol is required for each site to provide meaningful data.

The study population considered is made exclusively of healthy subjects with normal perfusion. Therefore, the generalizability of the findings to clinical populations or low-perfusion conditions remains to be investigated in future studies.

## 6. Conclusions

This study assessed the impact of the anatomical site and light wavelength on the quality and reliability of reflective-mode PPG signals acquired using two commercial sensors, including anatomical sites not previously investigated, and compared with the finger and wrist. The finger and wrist showed the best and worst signal qualities, respectively. The lips, nose, and temple yielded markedly superior signal quality and HR estimation compared with the wrist, and in several cases provided performance comparable to the finger, emphasizing their potential value as alternative acquisition sites.

The GREEN wavelength provided the strongest pulsatile component and the lowest HR estimation error across almost all anatomical sites, supporting its continued adoption in wearable technologies.

Future work should investigate these findings under dynamic conditions, including motion, thermal stress, and altered perfusion states, as well as in clinical populations. Practical aspects such as comfort and stability are critical for long-term monitoring. Facial sites such as the lips and temple offer relatively flat surfaces and stable sensor coupling; however, user acceptability and integration strategies (e.g., masks, glasses, protective equipment) must be carefully considered when designing wearable solutions.

Additionally, integrating real-time SQI monitoring and automatic site-adaptation strategies may further improve the robustness and reliability of reflective-mode PPG in practical wearable applications.

## Figures and Tables

**Table 1:** SQIs parameters at different anatomical sites for ADPD144RI sensor. Skewness (S) and Kurtosis (K) are dimensionless quantities, Perfusion Index (PI) is measured in %, Shannon Entropy (SE) in bits. Statistical significance: ^+^ *p* < 0.05 vs. finger, ^+++^ *p* < 0.005 vs. finger; * *p* < 0.05 vs. wrist, ** *p* < 0.01 vs. wrist, *** *p* < 0.005 vs. wrist.

Signal	SQI	Finger	Wrist	Ear	Nose	Temple	Lower Lip	Upper Lip
RED	S	0.74 ± 0.25 ***	0.08 ± 0.22 ^+++^	0.25 ± 0.21 ^+++^*	0.32 ± 0.20 ^+++^***	0.42 ± 0.25 ^+++^***	0.29 ± 0.23 ^+++^***	0.36 ± 0.26 ^+++^***
	K	2.90 ± 0.51 ***	2.53 ± 0.20 ^+++^	2.28 ± 0.19 ^+++^***	2.24 ± 0.23 ^+++^***	2.39 ± 0.27 ^+++^	2.32 ± 0.32 ^+++^*	2.42 ± 0.35 ^+++^*
	PI	3.53 ± 1.26	3.42 ± 1.06	3.85 ± 2.27	3.72 ± 1.64	3.97 ± 1.98	4.32 ± 1.96	4.08 ± 1.87
	SE	3.68 ± 0.16	3.71 ± 0.16	3.59 ± 0.24 *	3.73 ± 0.13	3.67 ± 0.17	3.62 ± 0.18	3.65 ± 0.24
IR	S	0.79 ± 0.29 ***	0.06 ± 0.18 ^+++^	0.29 ± 0.21 ^+++^***	0.31 ± 0.16 ^+++^***	0.41 ± 0.23 ^+++^***	0.33 ± 0.25 ^+++^***	0.33 ± 0.26 ^+++^***
	K	3.00 ± 0.64 ***	2.52 ± 0.23 ^+++^	2.31 ± 0.29 ^+++^***	2.26 ± 0.31 ^+++^***	2.43 ± 0.32^+++^	2.34 ± 0.25 ^+++^*	2.39 ± 0.41 ^+++^
	PI	4.13 ± 2.39	3.55 ± 1.37	3.57 ± 1.95	4.02 ± 1.07 *	4.42 ± 2.70	4.61 ± 2.74 *	3.98 ± 1.99
	SE	3.71 ± 0.14	3.71 ± 0.16	3.60 ± 0.25	3.61 ± 0.25	3.69 ± 0.19	3.59 ± 0.16 ^+^**	3.64 ± 0.21

**Table 2:** SQIs parameters at different anatomical sites for MAX30101 sensor. Skewness (S) and kurtosis (K) are dimensionless quantities; perfusion index (PI) is measured as %; Shannon entropy (SE) in bits. Statistical significance: ^+^ *p* < 0.05 vs. finger, ^++^ *p* < 0.01 vs. finger, ^+++^ *p* < 0.005 vs. finger; * *p* < 0.05 vs. wrist, ** *p* < 0.01 vs. wrist, *** *p* < 0.005 vs. wrist.

Signal	SQI	Finger	Wrist	Ear	Nose	Temple	Lower Lip	Upper Lip
RED	S	0.69 ± 0.24 ***	0.08 ± 0.15 ^+++^	0.22 ± 0.21 ^+++^**	0.33 ± 0.20 ^+++^***	0.29 ± 0.18 ^+++^***	0.20 ± 0.25 ^+++^	0.26 ± 0.25 ^+++^**
	K	2.80 ± 0.42 ***	2.45 ± 0.27 ^+++^	2.36 ± 0.23 ^+++^	2.28 ± 0.27 ^+++^*	2.23 ± 0.30 ^+++^***	2.31 ± 0.26 ^+++^	2.37 ± 0.24 ^+++^
	PI	3.11 ± 0.94 ***	2.82 ± 0.28 ^+++^	2.85 ± 0.23 ^+++^	2.86 ± 0.14 ^++^*	2.80 ± 0.14 ^+++^	2.92 ± 0.21 **	3.00 ± 0.39 ***
	SE	3.68 ± 0.13	3.60 ± 0.22	3.63 ± 0.17	3.60 ± 0.22	3.65 ± 0.13	3.59 ± 0.19 ^+^	3.56 ± 0.68
IR	S	0.75 ± 0.24 ***	0.12 ± 0.17 ^+++^	0.30 ± 0.21 ^+++^**	0.36 ± 0.20 ^+++^***	0.34 ± 0.20 ^+++^***	0.32 ± 0.24 ^+++^***	0.41 ± 0.22 ^+++^***
	K	2.89 ± 0.49 ***	2.49 ± 0.23 ^+++^	2.23 ± 0.29 ^+++^***	2.18 ± 0.30 ^+++^***	2.18 ± 0.35 ^+++^***	2.19 ± 0.28 ^+++^***	2.31 ± 0.43 ^+++^
	PI	3.65 ± 1.19 ***	2.87 ± 0.25 ^+++^	2.94 ± 0.16 ^+++^*	3.12 ± 0.20 ^+++^**	2.97 ± 0.26 ^+++^*	3.22 ± 0.45 ^++^***	3.34 ± 0.76 ^+^***
	SE	3.69 ± 0.12 *	3.60 ± 0.21 ^+^	3.66 ± 0.15	3.67 ± 0.14	3.66 ± 0.14	3.65 ± 0.14	3.57 ± 0.68
GREEN	S	0.71 ± 0.27 ***	0.22 ± 0.21 ^+++^	0.33 ± 0.16 ^+++^*	0.33 ± 0.15 ^+++^**	0.28 ± 0.15 ^+++^	0.35 ± 0.14 ^+++^***	0.39 ± 0.17 ^+++^***
	K	2.75 ± 0.57 ***	2.17 ± 0.31 ^+++^	2.07 ± 0.25 ^+++^	2.06 ± 0.29 ^+++^*	1.89 ± 0.20 ^+++^***	1.97 ± 0.20 ^+++^***	2.03 ± 0.29 ^+++^
	PI	5.64 ± 2.30 ***	3.25 ± 0.34 ^+++^	4.31 ± 0.95 ^++^***	5.97 ± 1.76 ***	4.72 ± 1.16 ***	5.76 ± 1.49 ***	5.28 ± 0.92 ***
	SE	3.64 ± 0.25	3.64 ± 0.14	3.70 ± 0.13	3.69 ± 0.11	3.70 ± 0.17 *	3.76 ± 0.10 **	3.70 ± 0.09

**Table 3:** Number of “wins” across all pairwise comparisons of the ADPD144RI.

ADPD144RI			
Anatomical Site	Skewness Wins (RED + IR)	PI Wins (RED + IR)	Kurtosis Wins (RED + IR)
Finger	12	0	0
Temple	4	0	2
Nose	2	2	6
Lower Lip	2	2	4
Ear	2	0	4
Upper Lip	2	0	3
Wrist	0	0	2

**Table 4:** Number of “wins” across all pairwise comparisons of the MAX30101.

MAX30101						
Anatomical Site	Skewness Wins (RED + IR)	PI Wins (RED + IR)	Kurtosis Wins (RED + IR)	Skewness Wins (GREEN)	PI Wins (GREEN)	Kurtosis Wins (GREEN)
Finger	12	10	0	6	2	0
Upper Lip	4	6	2	3	3	2
Nose	4	5	4	1	3	2
Lower Lip	1	5	3	2	3	3
Ear	2	1	3	1	1	1
Temple	2	1	6	0	1	5
Wrist	0	0	2	0	0	1

**Table 5:** Percentage errors for RED and IR signals of the ADPD144RI.

Anatomical Site	HR Error RED (%)	HR Error IR (%)
Temple	3.59 ± 2.07	3.18 ± 1.48
Upper Lip	3.95 ± 4.14	3.81 ± 3.47
Nose	4.73 ± 3.77	3.94 ± 3.47
Lower Lip	4.76 ± 4.06	4.69 ± 4.48
Ear	5.01 ± 5.68	5.15 ± 7.49
Finger	5.71 ± 6.10	6.07 ± 7.19
Wrist	14.31 ± 15.46	15.16 ± 16.98

**Table 6:** Percentage errors for RED, IR, and GREEN signals of the MAX30101.

Anatomical Site	HR Error RED (%)	HR Error IR (%)	HR Error GREEN (%)
Lower Lip	0.56 ± 1.14	2.83 ± 6.32	1.63 ± 2.93
Upper Lip	1.00 ± 2.62	2.13 ± 3.69	1.54 ± 3.65
Finger	1.06 ± 1.52	1.31 ± 2.20	0.89 ± 1.44
Ear	1.21 ± 2.44	2.43 ± 2.86	2.58 ± 4.08
Nose	1.23 ± 1.85	1.20 ± 2.05	1.82 ± 3.19
Temple	2.40 ± 5.84	1.90 ± 3.18	1.55 ± 4.33
Wrist	4.57 ± 9.15	8.45 ± 9.25	9.32 ± 7.92

**Table 7:** Mean HR percentage error as a function of skewness of GREEN signal.

Skewness Range (a. u.)	HR Error (%)
0–0.2	2.91 ± 7.35
0.2–0.4	1.33 ± 2.40
0.4–0.6	0.80 ± 2.01
0.6–0.8	1.08 ± 1.72
>0.8	0.72 ± 0.91

**Table 8:** Mean HR percentage error as a function of perfusion Index of GREEN signal.

Perfusion Index Range (%)	HR Error (%)
2–4	2.65 ± 6.64
4–6	0.91 ± 1.79
6–8	1.29 ± 2.16
>8	0.57 ± 0.81

**Table 9:** Mean HR percentage error as a function of kurtosis of GREEN signal.

Kurtosis Range	HR Error (%)
1.8–2.0	0.74 ± 1.40
2.0–2.2	2.07 ± 6.62
2.2–2.4	0.96 ± 1.46
2.4–2.6	2.23 ± 3.58
>2.6	3.43 ± 5.79

## Data Availability

The data presented in this study are available on request from the corresponding author.

## Abbreviations

The following abbreviations are used in this manuscript:

PPG	Photoplethysmography
HR	Heart Rate
PTT	Pulse Transit Time
SQI	Signal Quality Index
ECG	Electrocardiography
IR	Infrared
LED	Light Emitting Diode
BLE	Bluetooth Low Energy
Hz	Hertz
dB	Decibel
s	Second
mm	millimeter
AC	Alternative Current
DC	Direct Current
S/s	Samples per second
IBI	Inter Beat Interval
S	Skewness
K	Kurtosis
PI	Perfusion Index
SE	Shannon entropy
